# 2D-QSAR and 3D-QSAR/CoMSIA Studies on a Series of (*R*)-2-((2-(1*H*-Indol-2-yl)ethyl)amino)-1-Phenylethan-1-ol with Human β_3_-Adrenergic Activity

**DOI:** 10.3390/molecules22030404

**Published:** 2017-03-05

**Authors:** Gastón Apablaza, Luisa Montoya, Cesar Morales-Verdejo, Marco Mellado, Mauricio Cuellar, Carlos F. Lagos, Jorge Soto-Delgado, Hery Chung, Carlos David Pessoa-Mahana, Jaime Mella

**Affiliations:** 1 Instituto de Química y Bioquímica, Facultad de Ciencias, Universidad de Valparaíso, Av. Gran Bretaña 1111, Playa Ancha, Valparaíso 2360102, Chile; apablaza.gaston@gmail.com (G.A.); luisa.montoya.lara@gmail.com (L.M.); 2 Laboratorio de Bionanotecnología, Universidad Bernardo OHiggins, General Gana 1702, Santiago 8320000, Chile; camoralv@uc.cl; 3 Departamento de Química, Universidad Técnico Federico Santa María, Av. España 1680, Valparaíso 2390123, Chile; marco.mellado@postgrado.usm.cl; 4 Facultad de Farmacia, Universidad de Valparaíso, Av Gran Bretaña 1091, Valparaíso 2360102, Chile; mauricio.cuellar@uv.cl; 5 Department of Endocrinology, School of Medicine, Pontificia Universidad Católica de Chile, Lira 85, 5th Floor, Santiago 8330074, Chile; cflagos@uc.cl; 6 Facultad de Ciencia, Universidad San Sebastián, Campus Los Leones, Santiago 7510157, Chile; 7 Departamento de Ciencias Químicas, Facultad de Ciencias Exactas, Universidad Andrés Bello, Quillota 980, Viña del Mar 2531015, Chile; jorge.soto@unab.cl; 8 Pharmacy Department, Faculty of Chemistry, Pontificia Universidad Católica de Chile, Vicuña Mackenna 4860, Santiago 702843, Chile; chung.hery@gmail.com (H.C.); cpessoa@uc.cl (C.D.P.-M.)

**Keywords:** QSAR, CoMSIA, beta-3 adrenergic receptor, diabetes, obesity, mirabegron, vibegron, indole

## Abstract

The β_3_ adrenergic receptor is raising as an important drug target for the treatment of pathologies such as diabetes, obesity, depression, and cardiac diseases among others. Several attempts to obtain selective and high affinity ligands have been made. Currently, Mirabegron is the only available drug on the market that targets this receptor approved for the treatment of overactive bladder. However, the FDA (Food and Drug Administration) in USA and the MHRA (Medicines and Healthcare products Regulatory Agency) in UK have made reports of potentially life-threatening side effects associated with the administration of Mirabegron, casting doubts on the continuity of this compound. Therefore, it is of utmost importance to gather information for the rational design and synthesis of new β_3_ adrenergic ligands. Herein, we present the first combined 2D-QSAR (two-dimensional Quantitative Structure-Activity Relationship) and 3D-QSAR/CoMSIA (three-dimensional Quantitative Structure-Activity Relationship/Comparative Molecular Similarity Index Analysis) study on a series of potent β_3_ adrenergic agonists of indole-alkylamine structure. We found a series of changes that can be made in the steric, hydrogen-bond donor and acceptor, lipophilicity and molar refractivity properties of the compounds to generate new promising molecules. Finally, based on our analysis, a summary and a regiospecific description of the requirements for improving β_3_ adrenergic activity is given.

## 1. Introduction

Until 1967, only two classes of β-adrenergic receptors (β-ARs) were known, namely β_1_- and β_2_-AR [1,2,3]. However, by the 1980s a new class of β-AR [4] was found in several species, including bovine, rats and mice. Later, its presence was confirmed in humans and it was called β_3_-AR [5]. The three β-AR subtypes belong to the G-protein coupled receptors superfamily (GPCRs). The β_1_- and β_2_-ARs are located mainly in heart and lungs, respectively. The discovery of selective molecules that could specifically target one receptor subtype has prompted the modern treatment of hypertension and asthma. On the other hand, β_3_-AR has a wide tissue distribution, being present in adipose tissue [6], heart [7], detrusor muscle [8], bladder [9], prostate [10], gut [11], uterus [12], pancreas [13], and brain [14]. Accordingly, β_3_-AR ligands constitute potential drugs useful to treat several diseases [15]. For example, β_3_-AR agonists produce weight loss in obese animals without decreasing food intake [16]. They also seem to exert potent anti-diabetic effects in rodent models of type 2 diabetes [17], and chronic treatment with β_3_-AR agonists reduces hyperglycemia, hyperinsulinemia and hyperlipidemia in animal models [18]. In the rodent brain, the β_3_-AR agonist SR 58611A (Amibegron) displays an antidepressant profile [19], without side effects such as tachycardia or alteration of locomotor activity [20]. In failing hearts, the overall effect depends on the stage of the disease [21]. β_3_-AR agonists should serve in early stages because stimulation of β_3_-AR inhibits cardiac contractility, which counteracts the high plasma levels of catecholamines. In the late phase, however, highly selective antagonists/inverse agonists should be useful to improve the reduced cardiac contractility [22].

**Figure 1:**
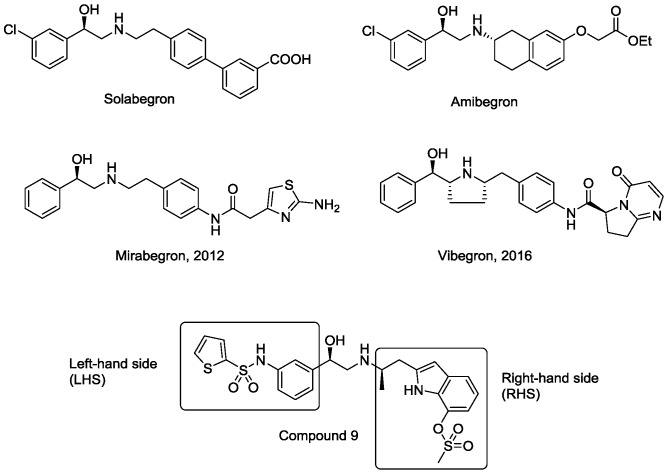
Structure of selected β_3_-adrenergic agonists and compound 9, the most potent of the analyzed series.

Many synthetic efforts have been carried out in order to obtain pharmacologically active agonists or antagonists. β_3_-AR agonists include phenylethanolamine molecules such as BRL 37344, GW-427353 (Solabegron), SR-58611A (Amibegron) [23], and YM-178 (Mirabegron) [24] (Figure 1), while antagonists include aryloxypropanolamines such as SR 59230A, L-748337 and CGP-20712A. Despite recent efforts for obtaining new derivatives with β_3_-AR affinity [25,26], Mirabegron has been the only drug approved by the FDA for the treatment of overactive bladder (OAB). However, in August 2015, the FDA raised concerns due to reported cases of life-threatening upper airway angioedema, which resulted from even first administration of Mirabegron. Likewise, in October 2015, the Medicines and Healthcare products regulatory agency (MHRA) of UK alerted the risk of severe hypertension and associated cerebrovascular and cardiac events. This has raised doubts on the future continuity of Mirabegron on the market. A potentially promising new molecule was reported by Merck earlier this year (Vibegron, 2016) [27] (Figure 1). Therefore, we must prioritize the formulation of structure–activity relationship models that can provide useful information for designing new compounds with improved affinity and selectivity for the β_3_-AR, as well as less side effects.

Until now, the human β_3_ receptor has not been crystallized and the design of compounds reported in literature is based on the random exploration of new fragments at the right-hand side (RHS) or left-hand side (LHS) of an ethanolamine core. In this context, the 3D-QSAR techniques become useful as tools to rationally design and direct the synthesis of potentially active derivatives. Few QSAR studies on this receptor have been reported in the literature [28,29,30]. Some of the limitations presented by these works include low predictability of the test set compounds [28], poor data distribution along the line y = x, a narrow range of studied biological activity [29], and the low potency of compounds used in the formulation of the model. Other studies do not demonstrate that the combination of the considered descriptors is optimal, which can lead to explanations of the structure–activity correlation based on nonsignificant information [30].

Our research group has been interested in the synthesis and QSAR studies of indoles and benzimidazoles with activity on GPCRs [31,32,33]. In this paper, we present a combined 2D- and 3D-QSAR study on a series of agonists of indolealkylamine structure with potent β_3_ adrenergic activity. A study was conducted using the CoMSIA technique [34], which allows us to explore the steric, electrostatic, hydrophobic, hydrogen-bond donor and hydrogen-bond acceptor field contributions of a series of compounds on their biological activity. Additionally, a Hansch analysis was carried out on the series, providing complementary information to the CoMSIA results. The information herein reported is summarized in a useful structure–activity relationship scheme for the design and synthesis of new indole type β_3_ adrenergic agonists. The best models were subjected to internal and external validation, obtaining good statistical parameters.

## 2. Results and Discussion

We carried out a step by step calculation of 31 models assaying all possible field combination with the aim to find the strongest models that contained the best set of descriptors. (Table 1). The best models were selected based on the highest *q^2^* values (0.639 and 0.626 for models 15 and 30, respectively). According to these models, the major contribution to biological activity is given by steric, hydrogen-bond donor and hydrogen-bond acceptor properties.

The predicted pEC_50_ for each compound and the residual values were calculated for the best models and for the 2D-QSAR equation (Table 2).

**Figure 2:**
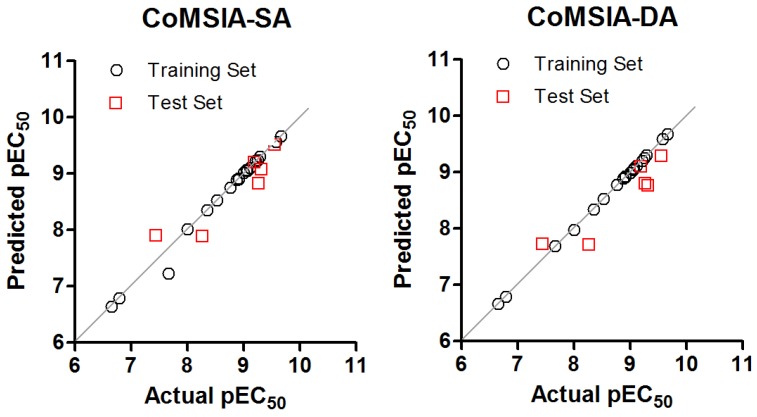
Graphics of Actual versus predicted pEC_50_ for models: 15 (**left**); and 30 (**right**).

The compounds were divided into a training set (19 compounds, 76%) and a test set (six compounds, 24%). The three models present a good linear fit and an adequate predictive power. The plot of actual versus predicted activities is depicted in Figure 2 for the CoMSIA models.

Unlike 2D-QSAR, the results of a CoMSIA study can be seen as contour maps around the surface of the studied compounds. This allows for an easier and more straightforward interpretation of the results. The steric, hydrogen-bond acceptor, and hydrogen-bond donor contour maps as well as the structure–activity relationships obtained from this analysis are presented below.

### 2.1. CoMSIA-SA (Model No.15)

**Figure 3:**
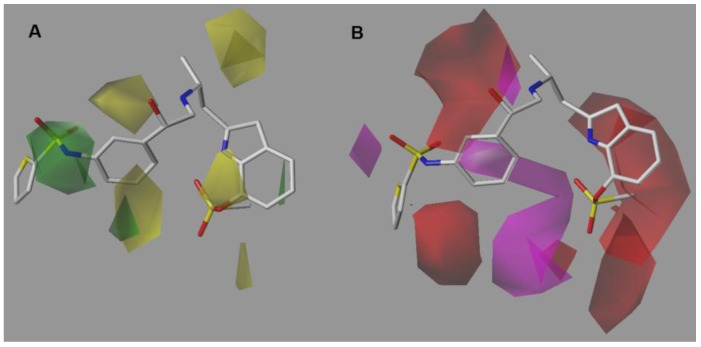
CoMSIA-SA model around compound **9**, the most potent of the series. (**A**) Steric contour map. Green contours indicate regions where bulky groups improve activity, whereas yellow contours indicate regions were bulky groups decreases activity. (**B**) Hydrogen-bond acceptor contour map. Magenta contours indicate regions where hydrogen-bond acceptor groups increase activity, whereas red contours indicate regions where hydrogen-bond acceptor groups decrease activity.

The steric contour map shows a green polyhedron in the LHS thiophene-sulfonamide group (Figure 3A). However, the thiophene ring is out the green polyhedron, so the use of short and bulky substituents would be appropriate in this position. To evaluate the importance of the thiophene ring, it would be interesting to explore the synthesis of derivatives only with the sulfonamide group but without the thiophene ring. Regarding the yellow contours, two yellow polyhedra are shown near positions 2 and 5 of the LHS benzene ring, suggesting that small or no substituents would be preferable on these sites. Similarly, the yellow polyhedra on the asymmetric carbon indicates that the insertion of bulky groups in this position is detrimental for activity. Finally, a yellow polyhedron on position 7 of the indole ring suggests that linkers smaller than a sulfonyl group could be used.

The hydrogen-bond acceptor contour map (Figure 3B) shows a big, restrictive, red polyhedron that is projected from position 2 of the LHS benzene ring. Therefore, the use of hydrogen-bond acceptors in that position is not favorable. Using halogens such as Cl, Br and I but not F would be favorable. The magenta polyhedron near the sulfate group of the indole ring indicates that another hydrogen-bond acceptor group could be used instead. For example, an ionizable group at physiological pH could be suitable since it is known that the presence of acids groups in the RHS is favorable for activity.

On the other hand, the spatial orientation of the sulfate group puts the methyl group inside a red polyhedron, which indicates the use of hydrogen-bond acceptors in that zone is not favorable. Additionally, the rotational freedom of the sulfate group seems to be restricted by a hydrogen bond between the oxygen atom of sulfate group and the proton of the indole ring, fixing it in a preferred orientation. Finally, the red polyhedron on the amine of the ethanolamine chain can be explained by the protonation state of this system inside cellular medium, which supports the need of a hydrogen-bond donor. The other magenta polyhedron near the oxygen atom of the LHS sulfonamide seems to indicate that this group could act as a hydrogen-bond acceptor and not only as a linker atom.

### 2.2. CoMSIA-DA (Model N°30)

**Figure 4:**
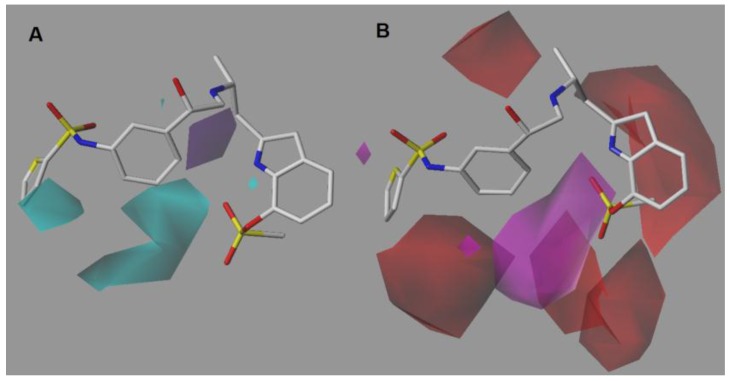
CoMSIA DA model around compound **9**, the most potent of the series. (**A**) Donor contour map. Cyan contours indicate regions where hydrogen-bond donors increase activity, whereas purple contours indicate regions where hydrogen-bond donors decrease activity. (**B**) Acceptor contour map. Colors have the same meaning as explained in Figure 3B.

The donor contour map (Figure 4A) displays a cyan polyhedron on position 3 of the thiophene ring. Therefore, the insertion of a hydrogen-bond donor such as NH2, OH and CONH2, would be favorable in this position. Likewise, in position 5 of the LHS benzene ring a big cyan contour indicates that addition of a hydrogen-bond donor is favorable for activity. On the other hand, a purple polyhedron on the methylene bridge connected to the indole ring restricts the use of hydrogen-bond donors in that position.

In general, the acceptor contour map (Figure 4B) is in agreement with the CoMSIA-SA model. A red polyhedron on the protonable nitrogen atom of the ethanolamine chain supports the hydrogen-bond donor required in that position. As for the sulfate group of the indole ring, the red and magenta polyhedra show the same disposition seen in the above model, highlighting that the presence of the oxygen atom in the sulfate group of the indole ring is favorable.

### 2.3. 2D-QSAR Model

**Figure 5:**
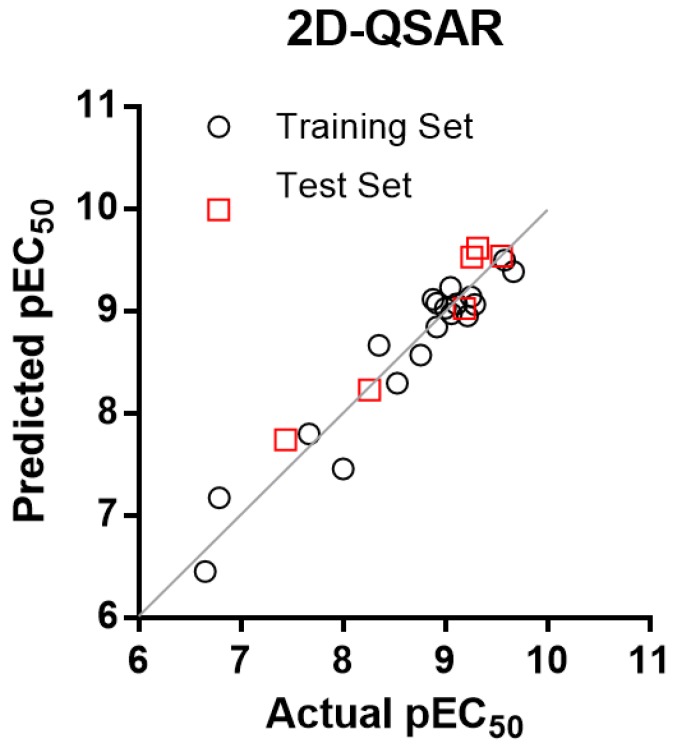
Graphic of actual against predicted pEC_50_ for Equation (3).

**Figure 6:**
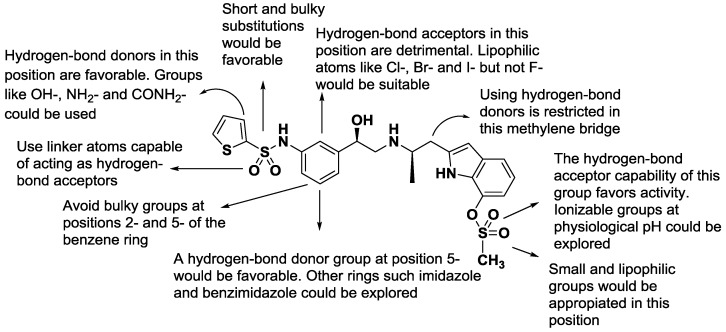
Structure–activity relationships derived from CoMSIA/Hansch studies.

A Hansch analysis was carried out in order to expand the SAR information for this class of compounds. After testing a wide number of descriptors and combinations between them, the best equation found was the following Equation (1):

(1) $$-\log EC_{50}=0.057\left(\pm 0.013\right)CMR^{2}-1.65\left(\pm 0.38\right)CMR+2.37\left(\pm 0.25\right)S+0.072\left(\pm 0.049\right)\pi_{x}^{2}+0.098\left(\pm 0.024\right)\pi_{y}^{2}+18.56$$

$$n=21,\;r^{2}=0.9072,\;F=29.32,\;p<10^{-6},\;s=0.321,\;q^{2}=0.607,\;SEP=0.524,\;N_{LOO}=5,\;{r^{2}}_{\mathrm{pred}}=0.9379$$

where CMR is the calculated molar refractivity, and S is a Free-Wilson parameter that describes the presence or absence of the LHS sulfonamide group independent of the cycle to which it is attached. πx is the lipophilicity of the substituent connected to position 7 of the indole ring, πy is the lipophilicity of the substituent connected to the LHS benzene ring with regardless of the position. From this equation it can be seen that the presence of a sulfonyl group is highly favorable for activity, as well as high lipophilicity of the substituents in the indole ring and LHS benzene ring. On the other hand, the biological activity decreases drastically with increasing molar refractivity, and therefore, the insertion of halogens such as bromine or iodine should be explored with caution. The plot of actual versus predicted activities is depicted in Figure 5 for the 2D-QSAR model. Figure 6 summarizes the principal structure-activity relationships found in this study that can serve as a basis for the development of new promising compounds.

## 3. Materials and Methods

### 3.1. Data Set Selection and β_3_ Adrenergic Activity

The CoMSIA and 2D-QSAR studies were performed on a set of 25 diverse molecules obtained from literature [35,36,37,38,39] with the general structure 2-alkylaminoindole (Table 3). The biological activity of the compounds was measured under the same laboratory and experimental conditions and was expressed as EC_50_. β-ARs agonistic activity was assessed by measuring cAMP accumulation in CHO cells expressing β_3_ receptors. The compounds displayed a high selectivity for the β_3_ receptor in the functional assays. The biological activity was converted to pEC_50_ (=−logEC_50_, in molar concentration). The compounds were randomly divided into training and test sets, ensuring that both sets contained structurally diverse compounds with high, medium and low activity, and a uniform distribution to avoid possible problems during the external validation. The first generation β_3_-AR agonist BRL-37344 was included in the test set (compound **3**).

### 3.2. Parameter Calculations and Statistical Analysis

For the 2D-QSAR study, the molar refractivity (CMR) and lipophilicity (CLogP) parameters were calculated using the ChemBioDraw software (15.1.0, PerkinElmer, Waltham, MA, USA). The multilinear regression analysis was performed with the Statistica Software (8.0, StatSoft, Tulsa, OK, USA). All the combinations among the independent variables were evaluated. The best model herein presented contains the fewest number of independent variables to avoid overfitting [40] and chance correlation [41] and to obtain the highest correlation coefficient. Internal validation of the model was carried out using the Leave-one-out method (LOO) which generated the crossvalidation regression coefficient (*q^2^*). The predictive power of the models was assessed by the calculation of *r^2^*_pred_ [42] as described below.

### 3.3. Selection of Conformers and Molecular Alignment

CoMSIA studies were performed with Sybyl-X software (1.2, Tripos International, St. Louis, MS, USA) [43] installed in a Windows 7 environment on a PC with an Intel core i7 CPU. In order to acquire the best conformers for each molecule, every compound was subjected to a preliminary geometry optimization of 1000 iterations using the Tripos force field implemented in Sybyl [44]. The convergence criterion of the energy gradient was set to 0.005 kcal/molÅ, and Gasteiger-Hückel charges were assigned to each atom [45], after which 10 cycles of simulated annealing dynamics were run heating the molecules to 1000 K for 1000 fs followed by the annealing of the compounds at 50 K for 1000 fs. From this analysis, the conformers with minimal total energy for each compound were chosen for the definitive CoMSIA studies. The minimized structures were superimposed by the atom fit method choosing the phenylethanolamine nucleus as the common scaffold for alignment.

### 3.4. CoMSIA Field Calculation

To derive the CoMSIA descriptor fields, the aligned training set molecules were placed in a 3D cubic lattice with grid spacing of 2 Å in x, y, and z directions such that the entire set was included in it. For CoMSIA analysis, the standard settings (probe with charge +1.0, radius 1 Å, hydrophobicity +1.0, hydrogen-bond donating +1.0, and hydrogen bond accepting +1.0) [34] were used to calculate five different fields: steric, electrostatic, hydrophobic, acceptor and donor. Gaussian-type distance dependence was used to measure the relative attenuation of the field position of each atom in the lattice. The default value of 0.3 was set for attenuation factor α.

### 3.5. Internal Validation and Partial Least Squares (PLS) Analysis

PLS analysis was used to construct a linear correlation between the CoMSIA descriptors (independent variables) and the activity values (dependent variables) [46]. To select the best model, the cross-validation analysis was performed by using the LOO method (and SAMPLS), which generates the square of the cross-validation coefficient (*q^2^*) and the optimum number of components (N). The non-cross-validation was performed with a column filter value of 2.0 in order to speed up the analysis and reduce the noise. The *q^2^*, which is a measure of the internal quality of the models, was obtained according to the following Equation (2):

(2) $$q^{2}=1-\frac{\sum {\left(y_{i}-y_{pred}\right)}^{2}}{\sum {\left(y_{i}-\bar{y}\right)}^{2}}$$

where $y_{i}$, $\bar{y}$, and $y_{pred}$ are observed, mean, and predicted activity in the training, respectively.

### 3.6. External Validation of the CoMSIA Model

The predictive power of the models was assessed by calculation of the predictive *r^2^* (*r^2^*_pred_) [42,47]. *r^2^*_pred_ measures the predictive performance of a PLS model and is defined according to Equation (3):

(3) $$r_{pred}^{2}=\frac{SD-PRESS}{SD}$$

where SD is the sum of the squared deviations between the biological activities of the test set compounds and mean activity of the training set compounds, and PRESS is the sum of squared deviations between observed and predicted activities of the test set compounds.

The plot of the predicted pEC_50_ values versus the experimental ones for CoMSIA analyses is also shown in Figure 2, in which most points are well distributed along the line y = x suggesting that the quality of the 3D-QSAR models is good.

To further ensure the external predictive power of our model we have implemented the validation criterion of Tropsha [48]:

(4) $$q^{2}>0.5$$

(5) $$r^{2}>0.6$$

(6) $$\frac{\left(r^{2}-r_{0}^{2}\right)}{r^{2}}<0.1\;or\;\frac{\left(r^{2}-r_{0}^{\prime 2}\right)}{r^{2}}<0.1$$

(7) $$0.85\leq k\leq 1.15\;or\;0.85\leq k^{\prime }\leq 1.15$$

where *q^2^* is the cross-validated correlation coefficient from LOO; *r^2^* is the correlation coefficient for experimental (*y*) vs. predicted (y*) activities for the test set molecules; *r_0_^2^* and *r'_0_^2^* are the correlation coefficients for the regression through origin for *y* vs. y* and y* vs. *y*, respectively; and *k* and *k'* are the slopes for regression through origin for y^r0^ = ky* and y*^r0^ = k'y. All of the models reported herein accomplish these criteria.

## 4. Conclusions

We have performed a CoMSIA study and a 2D-QSAR analysis from which valuable information can be obtained to direct the rational design and the synthesis of new β_3_-AR derivatives. The properties of the compounds found to correlate with the biological activity were: steric, hydrogen-bond donor and acceptor as well as lipophilicity and molar refractivity. The best models obtained presented good regression coefficients in internal and external validation. Finally, a proposed series of molecules is shown in Table 4 with their predicted biological activity. 

## Figures and Tables

**Table 1:** Sequential search for the generation of the best 3D-QSAR. Models ^a^.

Model No.	Model	*q^2^*	N	SEP	SEE	*r^2^*	*F*	Field Contributions				
								S	E	H	D	A
1	CoMSIA-S	0.393	1	0.668	0.432	0.746	47.005	1				
2	CoMSIA-E	0.414	9	0.928	0.004	1.000	75,589.127		1			
3	CoMSIA-H	0.493	9	0.863	0.007	1.000	26,900.499			1		
4	CoMSIA-D	0.251	8	0.989	0.02	1.000	3582.73				1	
5	CoMSIA-A	0.493	3	0.652	0.288	0.901	42.56					1
6	CoMSIA-SE	0.443	7	0.809	0.004	1.000	83,581.585	0.335	0.665			
7	CoMSIA-SEH	0.439	2	0..663	0.203	0.948	135.576	0.193	0.432	0.375		
8	CoMSIA-SEHD	0.437	10	0.973	0.000	1.000	5.92 × 10^6^	0.143	0.308	0.251	0.298	
9	CoMSIA-SEHA	0.530	2	0.607	0.190	0.954	155.022	0.135	0.285	0.258		0.322
10	CoMSIA-SED	0.448	4	0.707	0.037	0.998	2115.698	0.193	0.412		0.394	
11	CoMSIA-SEA	0.552	2	0.593	0.209	0.944	126.755	0.185	0.38			0.434
12	CoMSIA-SEDA	0.581	7	0.702	0.007	1.000	33,768.991	0.129	0.28		0.296	0.294
13	CoMSIA-SH	0.504	5	0.697	0.067	0.995	523.658	0.349		0.651		
14	CoMSIA-SD	0.356	10	1.04	0.005	1.000	56,928.168	0.342			0.658	
15	CoMSIA-SA	0.639	7	0.732	0.014	0.989	6334.553	0.387				0.613
16	CoMSIA-SHD	0.434	5	0.745	0.056	0.997	755.261	0.211		0.378	0.411	
17	CoMSIA-SHA	0.579	7	0.703	0.021	1.000	3754.265	0.217		0.385		0.398
18	CoMSIA-SDA	0.599	10	0.821	0.001	1.000	823,177.397	0.185			0.397	0.418
19	CoMSIA-SHDA	0.594	6	0.659	0.021	1.000	4613.236	0.137		0.251	0.3	0.312
20	CoMSIA-EH	0.421	2	0.674	0.216	0.941	118.677		0.533	0.467		
21	CoMSIA-ED	0.404	4	0.734	0.072	0.994	556.137		0.516		0.484	
22	CoMSIA-EA	0.517	2	0.615	0.24	0.926	94.322		0.46			0.54
23	CoMSIA-EHD	0.424	2	0.672	0.198	0.950	143.245		0.352	0.307	0.341
24	CoMSIA-EHA	0.512	2	0.619	0.205	0.947	132.798		0.328	0.299		0.373
25	CoMSIA-EDA	0.586	7	0.698	0.011	1.000	13,051.808		0.321		0.339	0.34
26	CoMSIA-EHDA	0.557	9	0.807	0.001	1.000	1.71 × 10^6^		0.248	0.214	0.272	0.266
27	CoMSIA-HD	0.408	2	0.681	0.248	0.921	87.997			0.487	0.513	
28	CoMSIA-HA	0.514	2	0.618	0.239	0.927	95.784			0.45		0.55
29	CoMSIA-HDA	0.596	5	0.63	0.051	0.997	907.891			0.289	0.35	0.361
30	CoMSIA-DA	0.626	8	0.813	0.003	1.000	154,958.201				0.492	0.508
31	CoMSIA-ALL	0.557	8	0.761	0.001	1.000	1.44 × 10^6^	0.103	0.225	0.192	0.242	0.239

^a^ *q^2^* = the square of the LOO cross-validation (CV) coefficient; N = the optimum number of components; SEP = standard error of prediction; SEE is the standard error of estimation of non CV analysis; *r^2^* is the square of the non CV coefficient; *F* is the *F*-test value. S, E, H, D and A are the steric, electrostatic, hydrophobic, hydrogen-bond donor and hydrogen-bond acceptor contributions, respectively.

**Table 2:** Experimental and predicted biological activity by the best CoMSIA models (Models 15 and 30) and the 2D-QSAR model.

Mol.	Actual pEC_50_ (M)	CoMSIA-SA		CoMSIA-DA		2D-QSAR	
		Predicted pE50 (M)	Residual	Predicted pE50 (M)	Residual	Predicted pE50 (M)	Residual
1 ^t^	8.260	7.890	0.37	7.607	0.65	8.229	0.03
2	6.650	6.637	0.01	6.664	0.01	6.453	0.20
3 ^t^	7.670	7.229	0.44	7.686	−0.02	7.798	−0.13
4	9.051	9.061	−0.01	9.041	−0.01	9.232	−0.18
5	9.252	9.241	0.01	9.254	0.00	9.141	0.11
6	9.108	9.107	0.00	9.117	0.01	9.068	0.04
7	8.879	8.887	−0.01	8.885	0.01	9.114	−0.23
8	8.759	8.757	0.00	8.773	0.01	8.567	0.19
9	9.678	9.668	0.00	9.673	0.00	9.385	0.29
10	9.222	9.225	−0.01	9.207	−0.01	8.951	0.27
11 ^t^	9.553	9.519	0.03	9.286	0.26	9.535	0.01
12	9.292	9.296	−0.01	9.296	0.01	9.064	0.23
13	9.060	9.058	0.00	9.065	0.01	8.973	0.09
14	9.585	9.568	0.01	9.593	0.01	9.499	0.08
15 ^t^	9.187	9.198	−0.01	9.101	0.09	9.023	0.17
16	8.921	8.917	0.00	8.913	−0.01	9.077	−0.16
17 ^t^	9.319	9.080	0.24	9.013	0.31	9.613	−0.29
18 ^t^	9.260	8.830	0.43	9.101	0.16	9.527	−0.27
19	6.790	6.790	0.00	6.790	0.00	7.173	−0.38
20	8.921	8.899	0.02	8.931	0.01	8.842	0.08
21	7.440	7.900	−0.46	7.603	−0.16	7.740	−0.30
22	8.000	8.019	−0.02	7.975	−0.03	7.455	0.54
23	8.530	8.532	0.00	8.532	0.00	8.295	0.23
24	8.350	8.356	−0.01	8.341	−0.01	8.666	−0.32
25	9.000	9.010	−0.01	8.989	−0.01	9.026	−0.03

^t^ test set compounds.

**Table 3:** Structure, biological activity and selectivity index of the studied compounds.

Entry	Structure	EC_50_ (nM) β_1_ (β_1_/β_3_) β_2_ (β_2_/β_3_) β_3_	pEC_50_ β_3_ (M)
1	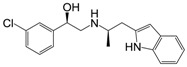	1.9 (0.4) 25 (4.6) 5.50	8.260
2	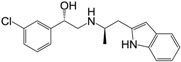	47 (0.2) 330 (1.5) 223	6.650
3	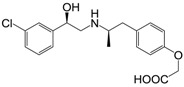	1700 (79.5) 290 (13.6) 21.38	7.670
4	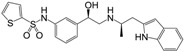	21 (23.6) 66 (74.2) 0.89	9.051
5	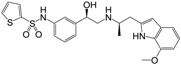	6.6 (11.8) 29 (51.8) 0.56	9.252
6	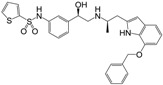	6.6 (8.5) 54 (69.2) 0.78	9.108
7	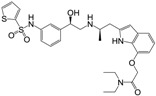	6.8 (5.2) 19 (14.4) 1.32	8.879
8	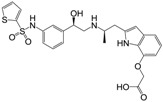	19 (10.9) 180 (103.4) 1.74	8.759
9	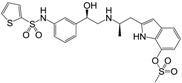	18 (85.7) 44 (19.1) 0.21	9.678
10	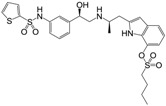	7.3 (12.2) 26 (43.3) 0.60	9.222
11	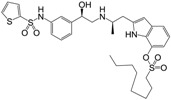	5.6 (20) 20 (71.4) 0.28	9.553
12	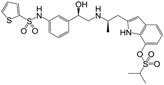	6.2 (12.2) 40 (78.4) 0.51	9.292
13	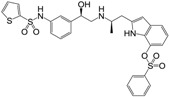	3.1 (3.6) 72 (82.8) 0.87	9.060
14	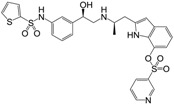	1.3 (5.0) 22 (84.6) 0.26	9.585
15	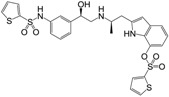	1.2 (1.8) 49 (75.4) 0.65	9.187
16	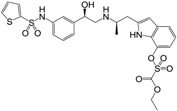	7.2 (6.0) 58 (48.3) 1.20	8.921
17	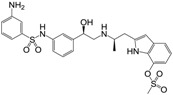	13 (27.1) 26 (54.2) 0.48	9.319
18	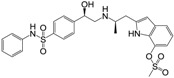	19 (34.5) 13 (23.6) 0.55	9.260
19	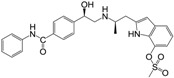	69 (0.43) 120 (0.74) 162	6.790
20	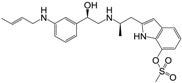	10 (8.3) 170 (141.7) 1.20	8.921
21	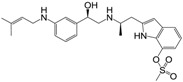	36 (1.0) 160 (4.4) 36.31	7.440
22	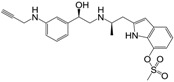	9.6 (1.0) 45 (4.5) 10.00	8.000
23	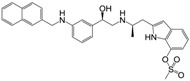	7.6 (25.8) 44 (14.9) 2.95	8.530
24	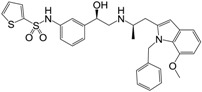	22 (4.9) 32 (7.2) 4.47	8.350
25	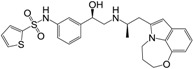	44 (44.0) 53 (53.0) 1.00	9.000

**Table 4:** The proposed structures of new molecules and their predicted pEC_50_ using the best model.

Entry	Structure	Predicted pEC_50_
QSAR_1	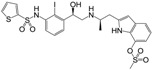	9.72
QSAR_2	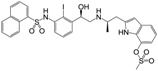	9.60
QSAR_3	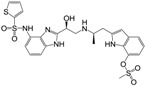	9.68
QSAR_4	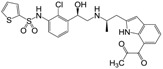	9.30
QSAR_5	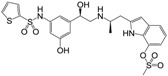	10.03
